# Patient-Reported Pain and Quality of Life Outcomes Following Iatrogenic Gallbladder Perforation During Laparoscopic Cholecystectomy

**DOI:** 10.14789/ejmj.JMJ25-0057-OA

**Published:** 2026-02-17

**Authors:** RANA BASIT ALI RAJPUT, ALI RAZA, MUHAMMAD WALEED KHAN, IRAM JAVAID KIANI, IQRA MAQBOOL RAJPUT, KHAULA RAFEEQ, MOHIT KUMAR

**Affiliations:** 1 Department of General Surgery, Combined Military Hospital, Lahore, Pakistan; 1 Department of General Surgery, Combined Military Hospital, Lahore, Pakistan; 2 Department of Trauma and Orthopaedics, Combined Military Hospital, Lahore, Pakistan; 2 Department of Trauma and Orthopaedics, Combined Military Hospital, Lahore, Pakistan; 3 Department of General Surgery, Combined Military Hospital, Lahore, Pakistan; 3 Department of General Surgery, Combined Military Hospital, Lahore, Pakistan; 4 Department of General Surgery, Umme Ilyas Hospital, Bangoain, Rawalakot, AJK, Pakistan; 4 Department of General Surgery, Umme Ilyas Hospital, Bangoain, Rawalakot, AJK, Pakistan; 5 Department of Medicine, Darulshafqat for Boys, Lahore, Pakistan; 5 Department of Medicine, Darulshafqat for Boys, Lahore, Pakistan; 6 Department of Medicine, Rural Medical College, Loni, Maharashtra, India; 6 Department of Medicine, Rural Medical College, Loni, Maharashtra, India

**Keywords:** laparoscopic cholecystectomy, iatrogenic gallbladder perforation, postoperative pain, Brief Pain Inventory, quality of life

## Abstract

**Objectives:**

Intraoperative gallbladder perforation (IGP) is a frequent adverse event in laparoscopic cholecystectomy; however, its influence on patient-reported pain and health-related quality of life (HRQoL) is underinvestigated. This research evaluated the level of postoperative pain and HRQoL in patients with and without IGP.

**Materials (Design):**

A longitudinal observational study was conducted on 300 adults who underwent elective laparoscopic cholecystectomy.

**Methods:**

The Brief Pain Inventory-Short Form (BPI-SF) and EQ-5D-5L assessed pain and HRQoL at 1 point in time (baseline) and at 2 points in time (3 and 6 months). Pearson's chi-square test, Mann-Whitney U test, Kruskal-Wallis test, and general linear models were used as statistical tests.

**Results:**

IGP was present in 80% of participants and was significantly associated with increased pain (r = 0.25, p < 0.001) and reduced HRQoL (r = -0.20, p < 0.001). HRQoL showed a moderate negative correlation with pain (r = -0.35, p = 0.001). Females complained about more pain and reduced QoL (p < 0.001). The IGP group also scored more in BPI-SF (baseline: 64.5 vs. 55.3; 6 months: 58.1 vs. 48.0) and continued to score lower in EQ-5D-5L. Even though pain and HRQoL improved over time, the difference between the IGP and non-IGP groups was substantial.

**Conclusion:**

Iatrogenic gallbladder perforation results in postoperative pain and a decrease in HRQoL that persisted up to six months postoperative. The use of preventive surgery and specific postoperative management is mandatory to enhance patient outcomes.

## Introduction

Laparoscopic cholecystectomy (LC) has become the standard treatment for acute cholecystitis due to gallstones, with advancements in technique and training making operations safer, and it has largely replaced open cholecystectomy^1, 2)^. Patients typically have short hospital stays (< 2 days) and rapid recovery, with low operative mortality (< 0.2%)^3)^.

Gallbladder perforation is an infrequent but serious complication associated with increased morbidity^4)^, occurring in 19-20% of laparoscopic cholecystectomies and often leading to more extended hospital stays, particularly in female patients^5, 6)^.

Preoperative factors, including higher QoL scores and shorter duration of pain, were associated with postoperative pain relief. At the same time, some studies report that gallbladder perforation during LC has no significant effect on respiratory performance, pain intensity, or hospital stay^7, 8)^.

Despite the widespread use of LC, postoperative quality of life remains poorly understood, with some studies reporting minimal or no improvement in QoL, particularly in asymptomatic patients^9, 10)^.

### Aims and objectives

The main objective of the research is to assess the effect of iatrogenic gallbladder perforation during LC on patient-perceived outcomes, specifically postoperative pain and QoL. The study aims to establish the existence of a difference in pain reported by patients who have undergone intraoperative perforation of the gallbladder compared to those who do not experience such a complication. Also, the research will investigate postoperative symptom timing and intensity, and include factors that can affect patient-reported outcomes and the general recovery pattern after this intraoperative procedure. Through patient-centred interventions, the research will aim to present evidence to inform perioperative counselling, optimise postoperative care, and develop strategies to reduce the negative impacts of gallbladder perforation, thereby improving the patient experience during laparoscopic cholecystectomy.

## Methods

### Study design and setting

This was a longitudinal observational study conducted to assess patient-reported pain and quality of life after laparoscopic cholecystectomy, including patients who experienced iatrogenic gallbladder perforation and those without intraoperative complications.

### Study population

Adult patients (18 years and above) who were to undergo elective laparoscopic cholecystectomy between January 2025 and July 2025 were recruited sequentially. The exclusion criteria were previous upper abdominal surgery, chronic pain disorder, cognitive impairment or a lack of informed consent.

### Sample size and technique

The population targeted was assumed to be effectively unlimited, since the number of adults who undergo laparoscopic cholecystectomy in Pakistan was not known. The minimum sample size was estimated at the 95% confidence level, with a 50% prevalence (to maximise sample size in the absence of local information) and a 5% margin of error. The result of this calculation was a minimum of 384 participants. A consecutive sampling method was also used, in which all qualified patients undergoing LC during the study period were invited to participate. To account for the possibility of non- response or non-follow-up, 400 patients were recruited first. The final sample, after applying inclusion and exclusion criteria and accounting for patient loss to follow-up, was 300. This reduction from the calculated sample size was due to patients not meeting eligibility criteria and incomplete follow-up data, which limited the number of participants available for final analysis.

### Data collection

Data were collected using three structured instruments, provided in their original English form without any language or cultural modifications. The following were the instruments:

### Demographic questionnaire

This questionnaire was completed at baseline to capture patient characteristics, including age, sex, body mass index, comorbidities, prior surgeries, and other pertinent clinical data. There was also a question of whether there was iatrogenic gallbladder perforation (IGP) when laparoscopic cholecystectomy was performed. Including this question in the demographic form enabled the standard documentation of the primary exposure variable (IGP) and baseline patient characteristics.

### Brief Pain Inventory (Short Form; BPI-SF)

The Brief Pain Inventory (Short Form; BPI-SF) is a validated measurement tool developed by Cleelen and Ryan in 1994 to assess the severity of pain and its interference with normal functioning. It is a 9-item scale, four of which measure pain intensity (worst, least, average, and current pain), and the remaining seven items ask about how pain disrupts general activity, mood, walking, work, interpersonal relationships, sleep, and enjoyment of life. Each of them is graded on a scale of 0 to 10, with 0 indicating the least pain or functional interference and 10 the greatest. Pain severity and pain interference are assessed using average scores^11)^. BPI-SF is selected for this study because it offers a more holistic, patient-centric evaluation of pain outcomes, including both pain intensity and the functional impact of pain, which is especially important in the period after complications such as iatrogenic gallbladder perforation.

### EQ-5D-5L Health Questionnaire

The EQ-5D-5L Health Questionnaire, developed in 1990 by the EuroQol Group and revised to a five-level version in 2009, is a widely used tool for measuring health-related quality of life (HRQoL). It measures five dimensions of health, including mobility, self-care, usual activities, pain/discomfort and anxiety/depression, with each dimension rated on a five-level scale of no problems to extreme problems. The questionnaire also includes a visual analogue scale (VAS) ranging from 0 (worst imaginable health) to 100 (best imaginable health) to reflect the patient's general opinion of their health condition. One index score can also be determined using sets of country-specific values, with higher scores indicating improved QoL^12)^. To assess longitudinal changes in overall health and HRQoL after surgery, the EQ-5D-5L was selected to compare outcomes between patients who underwent iatrogenic gallbladder perforation and those without surgical complications.

### Procedure

All participants were given the Brief Pain Inventory (Short Form; BPI-SF) and the EQ-5D-5L Health Questionnaire based on a predetermined order. Measurements on both instruments were performed at baseline (preoperatively) to assess baseline pain and QoL, and then again at 3 and 6 months after surgery. Follow-up Questionnaires were administered via face-to-face interviews conducted in the hospital or via structured telephonic interviews when in-person measures were not feasible. The questionnaires were administered using standardised instructions to ensure uniform understanding and proper completion. This schedule enabled tracking of longitudinal changes in pain intensity, pain interference, and overall quality of life, allowing comparison between patients with iatrogenic gallbladder perforation and those without intraoperative complications. To minimize bias, interviewers were trained using a standardized protocol, and follow-up assessments were conducted consistently at predefined time points.

### Statistical analysis

Baseline demographic and clinical characteristics were summarised using descriptive statistics. The relationships among iatrogenic gallbladder perforation (IGP), pain scores (BPI-SF), and quality of life (EQ-5D-5L) were evaluated using Pearson correlation analysis. Differences within gender groups were assessed using the Mann-Whitney U test, whereas differences across age categories were evaluated using the Kruskal-Wallis test. General linear models were used to test longitudinal changes in pain and quality of life, comparing pre- and post- follow-up to account for the repeated measures over time. All multivariate models were controlled for important confounders, including age, sex, comorbidities, and operative duration. The means and standard errors were used to summarise continuous variables, and the median was used, or the mean rank, to summarise non-parametric variables. The p-value was considered statistically significant if it was less than 0.05.

### Ethical considerations

The Institutional Review Board of Combined Military Hospital Lahore, Pakistan (CMH-LHR/ETH/25-0675) granted ethical approval, and the study proceeded in compliance with the Declaration of Helsinki. All participants provided written informed consent, and patient confidentiality was ensured.

## Results

### Demographic characteristics of the study participants (N = 300)

Table 1 indicates that the participants were mainly young adults, aged 18-25 years, with half in this bracket, and the sample had almost equal gender representation. Nearly half were married, and most had a primary or secondary education. The most common comorbidities were heart disease (23.3%) and respiratory disease (20%), but only 28.3% reported no comorbidities. The majority of the participants (65) had a history of abdominal surgery, and former smokers belonged to the biggest smoking group (45%). The primary cause of surgery was cholecystitis (58.3%), and 80% of the cases had IGP.

**Table 1:** Demographic characteristics of participants (N = 300)

Variable	f	%		Variable	f	%
Age				Comorbidities		
18-25 years	120	40.0		Diabetes	30	10.0
26-35 years	70	23.3		Hypertension	55	18.3
36-45 years	55	18.3		Heart disease	70	23.3
46-55 years	30	10.0		Respiratory disease	60	20.0
56-65 years	15	5.0		None	85	28.3
> 65 years	10	3.3		Previous abdominal surgeries		
Gender				Yes	195	65.0
Male	155	51.7		No	105	35.0
Female	145	48.3		Smoking status		
Marital status				Never smoked	90	30.0
Single	80	26.7		Former smoker	135	45.0
Married	140	46.7		Current smoker	75	25.0
Divorced	55	18.3		Reason for surgery		
Widowed	25	8.3		Gallstones	125	41.7
Educational level				Cholecystitis	175	58.3
No formal education	40	13.3		Iatrogenic Gallbladder Perforation (IGP)		
Primary school	85	28.3		Yes	240	80.0
Secondary/High school	95	31.7		No	60	20.0
College/University	55	18.3				
Postgraduate	25	8.3				

Note. N = number of participants; f = frequency, % = percentage

### Pearson correlations among iatrogenic gallbladder perforation, pain (BPI-SF), and QoL (EQ-5D-5L) (N = 300)

Table 2 reveals considerable correlations among IGP, pain, and quality of life. Higher pain scores were positively related to iatrogenic gallbladder perforation (r = 0.25, p = 0.001), which showed that patients with IGP had more postoperative pain. The IGP also showed a negative correlation with quality of life (r = -0.20, p = 0.001), indicating that the worse the QoL, the worse the perforation. The strongest association was observed in the table, with pain exhibiting a moderate negative relationship with QoL (r = -0.35, p = 0.001), such that higher pain levels were associated with lower QoL. In general, the results indicate that IGP and increased pain are associated with reduced postoperative QoL among such patients.

**Table 2:** Pearson correlations among iatrogenic gallbladder perforation (IGP), pain (BPI-SF), and quality of life (EQ-5D-5L) (N = 300)

Variable	1	2	3
Iatrogenic gallbladder perforation (IGP)	-	r = 0.25, t = 8.94, p = < 0.001***	r = -0.20, t = -7.06, p = < 0.001***
Brief pain inventory (BPI-SF)	-	-	r = -0.35, t = -12.93, p = < 0.001***
Quality of life (EQ-5D-5L)	-	-	-

Note. ***= p < 0.001 considered significant; Positive correlations indicate direct relationships, and negative correlations indicate inverse relationships; N = 300.

### Mann-Whitney U test comparing pain (BPI-SF) and quality of life (EQ-5D-5L) between male and female participants (N = 300)

Table 3 presents gender differences in pain and quality of life (QoL) assessed using the Mann-Whitney U test. Females reported significantly higher pain than males (Mean Rank = 169.00 vs. 130.79; U = 8,375.00, p < 0.001), while males reported higher QoL (Mean Rank = 167.21 vs. 132.29; U = 8,647.50, p = 0.001). These findings indicate that women experienced more postoperative pain and lower QoL compared to men.

**Table 3:** Mann-Whitney U test comparing pain (BPI-SF) and quality of life (EQ-5D-5L) by gender (N = 300)

Variable	Gender	N	Mean rank	Sum of ranks	U	Z	p
Brief pain inventory (BPI-SF)	Male	155	130.79	18,960.00	-	-	-
	Female	145	169.00	26,190.00	8,375.00	-4.80	<0.001***
Quality of life (EQ-5D-5L)	Male	155	167.21	25,917.50	-	-	-
	Female	145	132.29	19,232.50	8,647.50	3.25	0.001**

Note. N = 300 (Males = 155, 51.7%; Females = 145, 48.3%); Mann-Whitney U test was used for all comparisons; p values marked with **, *** indicate statistical significance at p < 0.01, < 0.001

### Mann-Whitney U test comparing pain (BPI-SF) and quality of life (EQ-5D-5L) by iatrogenic gallbladder perforation (IGP)

**Figure 1:**
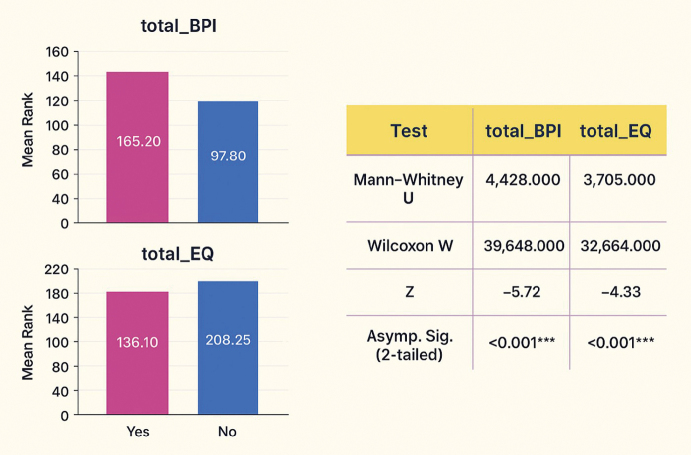
Mann-Whitney U test comparing pain (BPI-SF) and quality of life (EQ-5D-5L) by iatrogenic gallbladder perforation (IGP)

Figure 1 uses the Mann-Whitney U test to compare overall pain (BPI-SF) and quality of life (EQ-5D- 5L) between iatrogenic gallbladder perforation (IGP) and no-IGP patients. The level of pain was found to be significantly higher in patients with IGP, where the mean rank of 165.20 was much higher than it was in the patients who had not been perforated (U = 4,428, p < 0.001). QoL in patients with IGP, in contrast, was significantly lower, as indicated by a lower mean rank of 136.10 compared with 208.25 in the non-IGP group (U = 3,705, p < 0.001). The fact that both outcomes have negative Z scores shows that pain and QoL are worse in the IGP group, which agrees that perforation is related to a higher pain load and lower quality of life.

### Kruskal-Wallis test comparing pain (BPI-SF) and quality of life (EQ-5D-5L) across age groups (N = 300)

Table 4 indicates significant differences in both pain and quality of life across age groups. Younger participants (18-25 years) reported the highest pain levels, with mean ranks decreasing progressively with age (χ^2^(5) = 58.72, p < 0.001). Quality of life also declined with age, though the decrease was less steep (χ^2^(5) = 29.84, p < 0.001). Overall, older individuals experienced lower pain but also lower quality of life in the postoperative period, highlighting age-related variations in pain perception and well-being.

**Table 4:** Kruskal-Wallis test comparing pain (BPI-SF) and quality of life (EQ-5D-5L) across age groups (N = 300)

Variable	Age group	N	Mean rank	*x*^2^(df = 5)	p
Brief pain inventory (BPI-SF)	18-25 years	120	198.4	-	-
	26-35 years	70	176.2	-	-
	36-45 years	55	152.7	-	-
	46-55 years	30	128.5	-	-
	56-65 years	15	110.9	-	-
	>65 years	10	94.3	58.72	<0.001***
Quality of life (EQ-5D-5L)	18-25 years	120	187.6	-	-
	26-35 years	70	172.1	-	-
	36-45 years	55	158.9	-	-
	46-55 years	30	139.4	-	-
	56-65 years	15	121.7	-	-
	>65 years	10	105.2	29.84	<0.001***

Note. N = number of participants in each age category; % = percentage of the total sample; Percentages are based on total N = 300; Values are mean ranks from Kruskal-Wallis H tests; Overall test statistics are reported in the bottom row for each dependent variable: Total BPI-SF (χ^2^(5) = 58.72, < 0.001***) and Total ED-5D-5L (χ^2^(5) = 29.84, < 0.001***); Significance levels: p < 0.001***

### General linear model showing changes in pain (BPI-SF) and quality of life (EQ-5D-5L) over time by iatrogenic gallbladder perforation status (N = 2700)

Table 5 shows that patients who experienced iatrogenic gallbladder perforation (IGP) consistently reported higher pain and lower quality of life at all follow-up points compared to those without IGP. Pain decreased over time in both groups, yet IGP patients maintained significantly higher BPI-SF scores at baseline (64.5 vs. 55.3) and 6 months (58.1 vs. 48.0). Similarly, quality of life was lower in the IGP group, with baseline EQ-5D-5L scores of 15.9 versus 16.7 and 6-month scores of 16.2 versus 17.0, although both groups showed slight improvements. These findings indicate that IGP has a persistent negative impact on postoperative pain and overall quality of life.

**Table 5:** General linear model and mean pain (BPI-SF) and quality of life (EQ-5D-5L) scores over time by iatrogenic gallbladder perforation (N = 300)

Iatrogenic gallbladder perforation (IGP)	Brief pain inventory- timepoint	Mean	SE	df	95% CI lower	95% CI upper
Yes	Baseline	64.5	0.5	298	63.52	65.48
	3 months	61.2	0.5	298	60.22	62.18
	6 months	58.1	0.5	298	57.12	59.08
No	Baseline	55.3	0.5	298	54.32	56.28
	3 months	50.8	0.5	298	49.82	51.78
	6 months	48	0.5	298	47.02	48.98
Iatrogenic gallbladder perforation (IGP)	Quality of life- timepoint	Mean	SE	df	95% CI lower	95% CI upper
Yes	Baseline	15.9	0.1	298	15.7	16.1
	3 months	16.4	0.1	298	16.2	16.6
	6 months	16.2	0.1	298	16	16.4
No	Baseline	16.7	0.1	298	16.5	16.9
	3 months	16.4	0.1	298	16.2	16.6
	6 months	17	0.1	298	16.8	17.2

Note. SE = Standard Error; CI = 95% Confidence Interval. Differences between IGP groups are statistically significant at all time points.

## Discussion

This longitudinal study compared the effect of iatrogenic gallbladder perforation (IGP) on patient- reported pain and QoL after laparoscopic cholecystectomy. The higher observed frequency of iatrogenic gallbladder perforation in our cohort (80%) may be attributable to the institutional case mix, inclusion of minor perforations in the definition, and meticulous intraoperative documentation by surgeons. IGP was associated with higher pain scores. Likewise, past research documented that the postoperative pain of patients with perforated gallbladder was much more intense^13)^. Likewise, QoL in patients with IGP was worse, in line with the previous studies that postoperative complications such as ileus and site infections negatively influence well-being^14)^.

QoL in our study was moderately negatively related to pain (BPI-SF), consistent with previous research showing that higher pain levels are associated with lower EQ-5D scores^15)^. It also revealed gender differences, with females having higher pain scores and lower QoL than males, which is in line with previous results in surgical populations and in general population studies^16, 17)^.

Although our study revealed that IGP was associated with significantly increased pain levels, past research using visual pain scores did not find significant differences in postoperative pain between patients with and without gallbladder perforation, underscoring the need to use standardised patient- reported outcome measures^8)^. In our study, patients with iatrogenic gallbladder perforation (IGP) had significantly lower QoL than those without IGP. This is in line with the past research that indicated that postoperative complications in cholecystectomy have adverse impacts on the QoL of patients^18)^.

Age differences were also observed, with younger respondents experiencing greater pain and higher QoL, whereas older respondents reported less pain but lower QoL. These findings align with previous literature showing that pain sensitivity tends to decrease with age, particularly in modalities such as pressure pain. At the same time, health-related quality of life generally declines with age^19, 20)^.

In our research, patients with iatrogenic gallbladder perforation had higher pain scores at every time point, consistent with earlier research indicating higher postoperative pain in perforated cases. Still, our results demonstrate that this difference persists for up to 6 months^13)^. In line with longitudinal studies of HRQoL following laparoscopic cholecystectomy, our results indicate an increase in QoL over time. Still, patients with iatrogenic perforation consistently had lower QoL, suggesting the sustained effects of this intraoperative complication^21)^.

All in all, our results highlight the clinical significance of reducing iatrogenic gallbladder perforation during surgery, as it has long-term effects on postoperative pain and HRQoL. These impacts can only be captured through standardised patient- reported outcome measures, including BPI-SF and EQ-5D-5L.

There are limitations to the given study, such as a single-centre design and the use of English questionnaires, which might have led to similar problems in understanding the questions by some participants and introduced bias in the responses. The absence of intraoperative data (e.g., perforation size or surgeon experience) also limits a more in-depth explanation of the factors that affect outcomes in the postoperative period. Also, the 6-month follow- up can be inadequate to represent long-term recovery. Future studies ought to involve multicentre designs, use culturally sensitive instruments, collect comprehensive data on surgery, and follow up for at least 12 months. Additional effort is also needed to examine psychological variables that influence pain and compare specific postoperative interventions that could be utilised to enhance patient recovery in case of perforation of the gallbladder.

## Conclusion

In this study, it is shown that iatrogenic gallbladder perforation in laparoscopic cholecystectomy is strongly linked with increased postoperative pain and reduced QoL. These adverse effects last at least six months following the operation, and this implies that IGP was associated with long-term morbidity after the operation. Patient-reported outcomes were also associated with female gender and younger age. The results point to the necessity of perforation prevention through careful surgical practice, optimal postoperative care, and risk-specific patient follow-up. The reinforcement of the perioperative counselling efforts and introduction of the patient-centred management approaches can enhance recovery and overall well-being after laparoscopic cholecystectomy.

## Author contributions

RBR conceived and designed the study and supervised the overall project. AR contributed to the study design, patient recruitment, and interpretation of clinical outcomes. MWK assisted in data acquisition and the collection of hospital records. IJK contributed to surgical case assessment, interpretation of operative details, and clinical supervision. IMR supported data entry and quality checks and assisted with the literature review. KR performed statistical analysis, managed data, and contributed to table and figure formatting. MK contributed to the literature review, manuscript drafting, and assisted in organising referenced materials. All authors reviewed and approved the final manuscript. RBR served as the corresponding author.

## Conflicts of interest statement

The authors declare that there are no conflicts of interest.
